# Data monitoring committees in pediatric randomized controlled trials registered in ClinicalTrials.gov

**DOI:** 10.1177/17407745231182417

**Published:** 2023-06-27

**Authors:** Tiago Machado, Beatrice Mainoli, Daniel Caldeira, Joaquim J Ferreira, Ricardo M Fernandes

**Affiliations:** 1 Laboratory of Clinical Pharmacology and Therapeutics, Faculdade de Medicina, Universidade de Lisboa, Lisbon, Portugal; 2 Instituto de Medicina Molecular João Lobo Antunes, Lisbon, Portugal; 3 Clinical Research Unit, Research Center of IPO Porto (CI-IPOP), Porto, Portugal; 4 Campus Neurológico Sénior (CNS), Torres Vedras, Portugal; 5 Department of Pediatrics, Santa Maria Hospital, Centro Hospitalar Universitário Lisboa Norte, Lisbon, Portugal

**Keywords:** Clinical trials data monitoring committees, data and safety monitoring boards, pediatrics, clinical trials as topic

## Abstract

**Background:**

Data monitoring committees advise on clinical trial conduct through appraisal of emerging data to ensure participant safety and scientific integrity. While consideration of their use is recommended for trials performed with vulnerable populations, previous research has shown that data monitoring committees are reported infrequently in publications of pediatric randomized controlled trials. We aimed to assess the frequency of reported data monitoring committee adoption in ClinicalTrials.gov registry records and to examine the influence of key trial characteristics.

**Methods:**

We conducted a cross-sectional data analysis of all randomized controlled trials performed exclusively in a pediatric population and registered in ClinicalTrials.gov between 2008 and 2021. We used the Access to Aggregate Content of ClinicalTrials.gov database to retrieve publicly available information on trial characteristics and data on safety results. Abstracted data included reported trial design and conduct parameters, population and intervention characteristics, reasons for prematurely halting, serious adverse events, and mortality outcomes. We performed descriptive analyses on the collected data and explored the influence of clinical, methodological, and operational trial characteristics on the reported adoption of data monitoring committees.

**Results:**

We identified 13,928 pediatric randomized controlled trial records, of which 39.7% reported adopting a data monitoring committee, 49.0% reported not adopting a data monitoring committee, and 11.3% did not answer on this item. While the number of registered pediatric trials has been increasing since 2008, we found no clear time trend in the reported adoption of data monitoring committees. Data monitoring committees were more common in multicenter trials (50.6% vs 36.9% for single-center), multinational trials (60.2% vs 38.7% for single-country), National Institutes of Health–funded (60.3% vs 40.1% for industry-funded or 37.5% for other funders), and placebo-controlled (47.6% vs 37.5% for other types of control groups). Data monitoring committees were also more common among trials enrolling younger participants, trials employing blinding techniques, and larger trials. Data monitoring committees were more common in trials with at least one serious adverse event (52.6% vs 38.4% for those without) as well as for trials with reported deaths (70.3% vs 38.9% for trials without reported deaths). In all, 4.9% were listed as halted prematurely, most often due to low accrual rates. Trials with a data monitoring committee were more often halted for reasons related to scientific data than trials without a data monitoring committee (15.7% vs 7.3%).

**Conclusion:**

According to registry records, the use of data monitoring committees in pediatric randomized controlled trials was more frequent than previously reported in reviews of published trial reports. The use of data monitoring committees varied across key clinical and trial characteristics based on which their use is recommended. Data monitoring committees may still be underutilized in pediatric trials, and reporting of this item could be improved.

## Introduction

A data monitoring committee (DMC), also called a data safety monitoring board, is a group of independent experts assigned to advise on clinical trial conduct through risk-benefit appraisal of emerging data to ensure participant safety and scientific integrity.^1^ This group is usually composed of medical and biostatistics experts, free of conflicts of interest regarding the outcomes of the particular trial, who should have unrestricted access to unblinded ongoing results data in order to allow informed judgments and unbiased decisions regarding the trial continuation.^2^

Even though safety and efficacy data should be regularly monitored in all clinical trials, not all trials need the formal establishment of a DMC. The decision to implement a DMC should be taken on a case-by-case basis, taking into consideration key clinical and methodological characteristics. Both the US Food and Drug Administration (FDA) and the European Medicines Agency provide guidance on the implementation of DMCs and recommend that they be considered for trials performed in potentially vulnerable populations such as children.^3,4^ International networks of experts have provided guidance on this topic specifically for the pediatric population. The Star Child Health recommendations on DMCs specify key clinical and methodological characteristics of trials in pediatric populations that generally mandate a DMC such as trials investigating new interventions with few safety data, trials addressing major morbidity or mortality endpoints, trials on high-risk populations, trials with planned interim analysis with the possibility of early stopping, and trials with large sample sizes and multicenter trials.^5^

Previous research has shown that DMCs are reported infrequently in publications of pediatric randomized controlled trials (RCTs). In 2009, Fernandes et al. evaluated the frequency and quality of reporting on DMC composition and roles, interim analysis and early stopping in a sample of 648 pediatric trials published in high-impact journals. In this sample, only 17% of trial reports provided any details on DMCs, interim analysis, or early stopping.^6^ Gates et al.^7^ reviewed a randomly selected sample of 300 pediatric trials from the Cochrane Central Register of Controlled Trials, reaching similar conclusions: the reporting on DMC was infrequent (18%), even though it was more common in trials which characteristics may suggest that DMC implementation may be warranted such as trials with drug interventions or multicentric trials.

Since the adoption of the Food and Drugs Administration Amendments Act (FDAAA),^8^ in September 2007, clinical trial registration is required for all “applicable clinical trials” in the United States. This law stipulated that all non-phase I trials of FDA-regulated drugs and biologics, as well as non-feasibility trials of FDA-regulated devices, were required to report to ClinicalTrials.gov. Similarly, since 2004, all trials of any medicinal product conducted in the European Union are required to report to the European Union Clinical Trials Register or other approved registries such as ClinicalTrials.gov.

The aim of this study was to assess the frequency of DMC adoption according to ClinicalTrials.gov registry records of pediatric RCTs and examine the influence of some key trial characteristics on DMC implementation.

## Methods

We conducted a cross-sectional data analysis of all RCTs registered in ClinicalTrials.gov between 2008 and 2021 and performed exclusively in a pediatric population. An initial selection of trial records was performed using the advanced search function in ClinicalTrials.gov. All publicly available data on the initial selection of trials were retrieved using the Access to Aggregate Content of ClinicalTrials.gov (AACT) database. AACT is a publicly available relational database that contains all publicly available information concerning studies registered in ClinicalTrials.gov, including protocol and data on safety and efficacy results.^9^ Research ethics approval was not required as all used data are publicly available and only meta-data were used. Our dataset is made available as supplementary material.

### Eligibility criteria

We considered eligible any clinical trial record first registered on ClinicalTrials.gov between 1 January 2008 and 31 December 2021, reporting to apply randomization techniques, and with a maximum age for participant inclusion equal to or lower than 18 years. We restricted our analysis to trials first registered since 2008 to coincide with the adoption of the FDAAA in September 2007.

### Search strategy and trial selection

On 24 January 2022, we searched on ClinicalTrials.gov for studies registered between 2008 and 2021, listed as an “Interventional Study (Clinical Trial)” (regarding the “Study type” item) and including pediatric participants, independently of inclusion of adults. The search results were extracted into a spreadsheet. RStudio was used to directly access AACT and download all publicly available information on the initially identified studies.^10^ We then excluded any studies that were not listed as “Randomized” (regarding the “Allocation” item) or were listed as “Single Group Assignment” (regarding the “Intervention Model” item). Finally, we excluded trials for which the maximum age for inclusion was higher than 18 years.

### Data extraction and classification

For each selected trial record, we extracted the data on items regarding DMC implementation, on key clinical and methodological characteristics likely to influence the decision to use a DMC (e.g. trial phase, number of centers, blinding, and age of participants), and trial results that may be surrogates for the identification of high-risk trials (i.e. reported number of participants with serious adverse events (SAEs) and number of deaths due to any cause). Two authors independently categorized the reasons for the termination or suspension of trials according to a chart adapted from previous research on this topic.^11^ We used the definitions for data elements in ClinicalTrials.gov according to the Glossary of Common Site Terms,^12^ ClinicalTrials.gov Protocol Data Element Definitions,^13^ and ClinicalTrials.gov Results Data Element Definitions for Interventional and Observational Studies.^14^ We further categorized some of the data elements for the purposes of our analysis. Eligibility age limits were categorized into age groups according to the definitions developed by the National Institute of Child Health and Human Development.^15^ Supplemental Table S1 describes additional details regarding ClinicalTrials.gov extracted data elements definitions and adjustments made.

### Data analysis

We performed a descriptive analysis of the collected data. Trial characteristics were summarized descriptively using counts and percentages for categorical variables and median and interquartile range (IQR) (Q1–Q3) for continuous variables. Trial records not answering on DMC implementation were not excluded from summarizations. Other missing variables were treated likewise and are summarized alongside the results. We used Microsoft Excel and RStudio for data analysis and visualizations.

## Results

**Figure 1.**
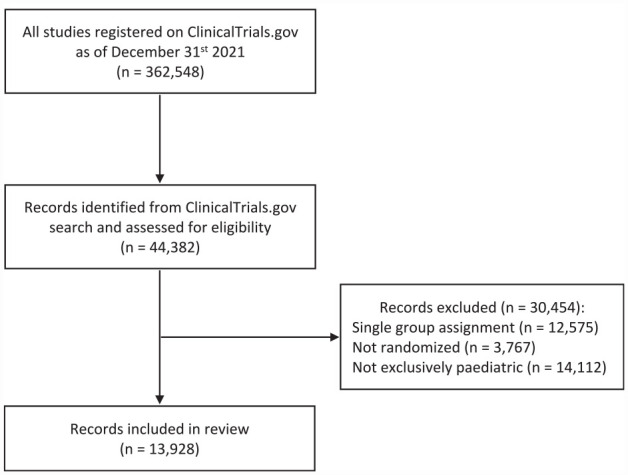
Flow diagram of records identification.

Our initial search query yielded 44,382 trial records. After the exclusion of single-group studies, non-randomized trials, and trials with adults, we included in the review 13,928 RCTs performed in an exclusively pediatric population (Figure 1).

### Trials characteristics

Trial characteristics, alongside DMC adoption rates, are presented in Table 1 and Supplemental Tables S2–S5. The number of registered RCTs in a pediatric population has been steadily increasing during the period under review: from 655 trials registered in 2008 to 1408 trials in 2021 (Supplemental Figure S1 and Table S3). Most trials (7817; 56.1%) were listed as completed, 3534 (25.4%) were ongoing, 682 (4.9%) were halted prematurely, and 348 (2.5%) were withdrawn before enrolling participants.

**Table 1.** Reported adoption of a DMC stratified by trial characteristics.

Trial characteristics	Total, n (%)	DMC, n (%)		
		DMC adoption	No DMC adoption	No answer regarding DMC
All included trials	13,928	5529 (39.7%)	6827 (49.0%)	1572 (11.3%)
Completion status				
Completed	7817 (56.1%)	2961 (37.9%)	4032 (51.6%)	824 (10.5%)
Ongoing	3534 (25.4%)	1478 (41.8%)	1563 (44.2%)	493 (14%)
Halted prematurely	682 (4.9%)	313 (45.9%)	327 (47.9%)	42 (6.2%)
Withdrawn	348 (2.5%)	153 (44%)	162 (46.6%)	33 (9.5%)
Unknown status	1547 (11.1%)	624 (40.3%)	743 (48%)	180 (11.6%)
Study sites (number of countries)				
Multinational	1040 (7.5%)	626 (60.2%)	277 (26.6%)	137 (13.2%)
Single country	11,252 (80.8%)	4359 (38.7%)	5727 (50.9%)	1166 (10.4%)
No data on study sites	1636 (11.7%)	544 (33.3%)	823 (50.3%)	269 (16.4%)
Study sites (number of sites)				
Multicentric	3293 (23.6%)	1667 (50.6%)	1297 (39.4%)	329 (10.0%)
Single center	8999 (64.6%)	3318 (36.9%)	4707 (52.3%)	974 (10.8%)
No data on study sites	1636 (11.7%)	544 (33.3%)	823 (50.3%)	269 (16.4%)
Clinical research phase				
Phase I	436 (3.1%)	216 (49.5%)	175 (40.1%)	45 (10.3%)
Phase II	1224 (8.8%)	695 (56.8%)	395 (32.3%)	134 (10.9%)
Phase III	1775 (12.7%)	929 (52.3%)	622 (35%)	224 (12.6%)
Phase IV	1387 (10.0%)	567 (40.9%)	653 (47.1%)	167 (12.0%)
Multiphase	621 (4.5%)	371 (59.7%)	194 (31.2%)	56 (9.0%)
Not applicable	8485 (60.9%)	2751 (32.4%)	4788 (56.4%)	946 (11.1%)
Sample size				
<100	7312 (52.5%)	2685 (36.7%)	3754 (51.3%)	873 (11.9%)
≥100	6616 (47.5%)	2844 (43.0%)	3073 (46.4%)	699 (10.6%)
Funding				
Industry	2605 (18.7%)	1044 (40.1%)	1196 (45.9%)	365 (14%)
National Institutes of Health	1063 (7.6%)	641 (60.3%)	349 (32.8%)	73 (6.9%)
Other	10,260 (73.7%)	3844 (37.5%)	5282 (51.5%)	1134 (11.1%)
Age groups^a^				
Newborns (0–27 days)	2623 (18.8%)	1235 (47.1%)	1165 (44.4%)	223 (8.5%)
Infants (28 days–12 months)	4019 (28.9%)	1753 (43.6%)	1832 (45.6%)	434 (10.8%)
Toddlers (12 months–2 years)	3253 (23.4%)	1390 (42.7%)	1492 (45.9%)	371 (11.4%)
2- to 5-year-old children	6127 (44.0%)	2429 (39.6%)	2956 (48.2%)	742 (12.1%)
6- to 11-year-old children	7651 (54.9%)	2917 (38.1%)	3847 (50.3%)	887 (11.6%)
12- to 18-year-old	5899 (42.4%)	2342 (39.7%)	2971 (50.4%)	586 (9.9%)
Type of control				
Placebo-controlled trials	3043 (21.8%)	1448 (47.6%)	1301 (42.8%)	294 (9.7%)
Not placebo-controlled trials	10,885 (78.2%)	4081 (37.5%)	5526 (50.8%)	1278 (11.7%)

DMC: data monitoring committee.

a Non-mutually exclusive groups.

The median number of participants per trial was 90 (IQR: 42–220). More than one country was involved in 1040 (7.5%) trials, and 3293 (23.6%) trials were multicenter. Most records reported that clinical research phase classification was not applicable (8485; 60.9%). According to the listed sponsors and collaborators, 2605 (18.7%) were funded by the industry, 1063 (7.6%) by the National Institutes of Health (NIH), and 10,260 (73.7%) by other sponsors. Nervous system diseases (2315; 16.6%) and mental disorders (1834; 13.2%) were the therapeutic areas more often studied (Supplemental Table S4). Children aged 6- to 11-year-old were eligible for 55% (7651) of the trials. In contrast, trials that included newborns represented 18.8% of our sample. Trials with drug interventions were the most common (4299; 30.9%), followed by behavioral interventions (3193; 22.9%) and devices (1573; 11.3%). The involved party more often blinded to the allocated intervention were the participants (6320; 45.4%), while 4727 (33.9%) trials did not employ any blinding techniques.

The vast majority (11,933; 85.7%) of the trials included in our review did not submit safety results data on the registry. Most trials that reported safety results were registered in the first half of the 14-year period under review (1423; 71.4%). For those reporting these results (1995; 14.3%), 44.6% (889) reported at least one SAE and 7.3% (145) reported at least one death due to any cause during the study.

### DMC adoption

Overall, a total of 5529 (39.7%) trials reported adopting a DMC, 6827 (49.0%) reported not adopting a DMC, whereas 1572 (11.3%) of trial records did not answer on the item on DMC adoption. DMC adoption was more common in trials that were halted prematurely (45.9%) than in completed trials (37.9%). DMC adoption was also more common in multicenter trials (50.6%) and multinational trials (60.2%). Trials enrolling or planning to enroll 100 participants or more commonly reported a DMC (43.0%) than smaller trials (36.7%), and the median number of participants per trial was higher for trials with a DMC (100; IQR: 46–250 vs 84; IQR: 40–200) (Supplemental Figure S2).

Phase II (56.8%) and multiphase trials (59.7%) had the highest rates of DMC adoption, while phase IV (40.9%) and trials for which the clinical research phase was listed as not applicable (32.4%) had the lowest rates. Trials funded by the NIH more frequently adopted a DMC (60.3%) than those funded by industry (40.1%) or trials with other sources of funding (37.5%).

DMCs were more common among trials enrolling younger participants; trials with newborns had the highest adoption rate (47.1%). Regarding the therapeutic areas of the disease or condition being studied (Supplemental Table S4), trials on neoplasms and cardiovascular diseases had the highest adoption rates, 57.3% and 57.4%, respectively. DMC adoption was more likely for trials testing drugs (50.2%), combination products (52.3%), and genetic interventions (75.0%), in contrast with trials testing behavioral interventions (32.7%) or diagnostic tests (26.5%) for which we measured the lowest rates. Trials reporting “treatment” as the main purpose of the intervention more frequently established a DMC (43.1%) compared to trials with other purposes. Placebo-controlled trials reported higher DMC adoption (47.6%) than trials with other types of control groups (37.5%). Overall, trials employing blinding techniques more frequently adopted a DMC (Supplemental Table S5), nevertheless, 33.7% of trials not employing any blinding did report using a DMC.

Trials reporting the occurrence of at least one SAE more frequently used a DMC (52.6%) than trials reporting no SAEs (38.4%). Similarly, trials reporting at least one death occurring during the study report higher DMC adoption rates (70.3%) than those without any deaths (38.9%; Table 2).

**Table 2.** Reported adoption of a DMC stratified by reporting of SAEs and mortality.

Safety results	Total, n (%)	DMC, n (%)		
		DMC adoption	No DMC adoption	No answer
SAEs				
Reports at least one SAE	889 (6.4%)	468 (52.6%)	262 (29.5%)	159 (17.9%)
No SAE reported	1106 (7.9%)	425 (38.4%)	620 (56.1%)	61 (5.5%)
No safety results posted	11,933 (85.7%)	4636 (38.9%)	5945 (49.8%)	1352 (11.3%)
All cause mortality				
Reports at least one death	145 (1.0%)	102 (70.3%)	22 (15.2%)	21 (14.5%)
No deaths reported	1850 (13.3%)	791 (42.8%)	860 (46.5%)	199 (10.8%)
No safety results posted	11,933 (85.7%)	4636 (38.9%)	5945 (49.8%)	1352 (11.3%)

DMC: data monitoring committee; SAEs: serious adverse events.

**Figure 2.**
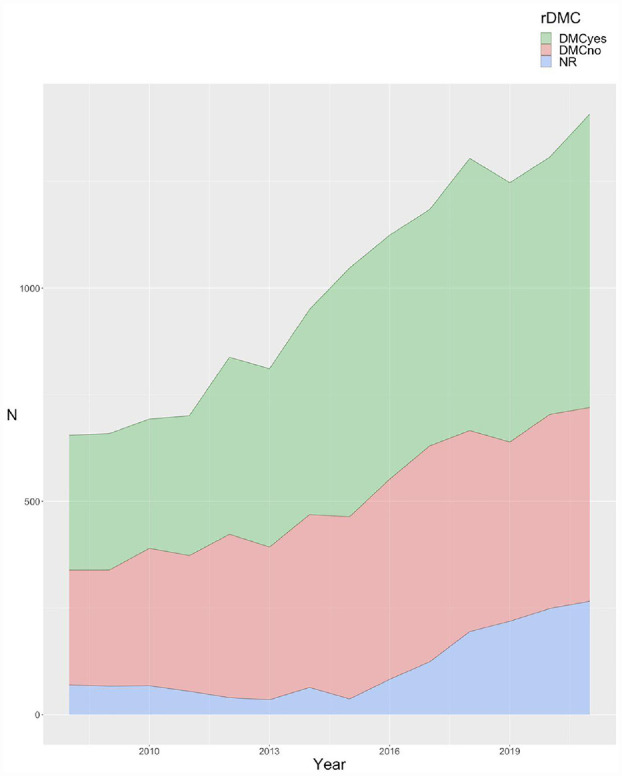
Reported adoption of a DMC per year.

We found no clear time trend in the adoption of DMCs. The rate of DMC adoption peaked at 45.7% in 2012, whereas in 2021 it reached the lowest value for the observed period (32.2%). This decline may be partially explained by the higher rates (15.0%–19.1%) of records not answering the DMC adoption item since 2018 (Figure 2 and Supplemental Table S3).

When focusing on a specific subset of trials of greater relevance for regulatory approval, that is, double-blind multicentric phase III trials, of 626 (4.5%) identified trials, 371 (59.3%) reported using a DMC.

### Reasons for prematurely halting

A total of 682 trials (4.9%) were listed as halted prematurely, either terminated (628) or suspended (55). Most reasons provided for stopping (Supplemental Table S5) were unrelated to scientific data from the trial (81.4%), most often due to a low accrual rate (40.6%). On the contrary, 11.4% were stopped for reasons related to scientific data from the trial, of which 70.5% denoted an unfavorable benefit-to-risk ratio (including safety issues, lack of efficacy, or futility), 7.7% mentioned a favorable benefit-to-risk ratio, while 21.8% were stopped for unspecified or other scientific data from the trial. Trials with a DMC were more often halted for reasons related to scientific data than trials without a DMC (15.7% vs 7.3%).

## Discussion

We conducted a cross-sectional data analysis of all pediatric RCTs registered in ClinicalTrials.gov between 2008 and 2021. To our knowledge, this is the largest study assessing DMC use in pediatric trials. We found that nearly 40% of pediatric RCTs reported having appointed a DMC, a higher estimate than in published trial reports, according to previous reviews.^6,7^ We found no clear time trend on the frequency of DMCs for the period under review, although the increasing use of DMCs over time has been previously suggested.^6^ Larger, multicenter, and multinational trials reported a higher frequency of DMC adoption, as did trials that employed blinding techniques, trials in younger age groups, and trials with drug or biological interventions. While some of these methodological and clinical features have been suggested as guides for special consideration of use of DMCs in pediatric trials,^5^ we found considerable heterogeneity in the adoption of DMCs based on these parameters.

There is a general consensus that decisions to adopt a DMC should be taken on a case-by-case basis, but specific characteristics need to be taken into account. Trial design and operational features that identify larger and more complex trials conducted across centers and countries are easily measured. Trials with these characteristics expectedly reported more frequent use of DMCs in our sample. Conversely, dimensions, such as the vulnerability of the eligible population, the severity of the condition, known and potential safety risks associated with interventions of interest, and the range of potentially severe outcomes, may be decisive features but are harder to capture. Among the available data elements, we chose the incidence of SAE or all cause mortality as surrogates for the identification of higher-risk trials. Despite the paucity of data on safety results, we found it reassuring that those trials with reported deaths or SAEs were more likely to have implemented a DMC.

Pediatric trials have added challenges that may influence the decision to adopt a DMC. Ethical considerations are particularly important when conducting clinical research with children, as their participation requires careful balance between the potential risks and benefits that vary based on age, context, and study characteristics. Our results suggest that the adoption of DMC is higher in younger age groups and contexts of greater morbidity and mortality. Furthermore, testing of new or repurposed medicines on de novo pediatric age ranges often needs to consider background information on efficacy and safety that may be borrowed or extrapolated from studies conducted in adults or children, or based on existing off-label pediatric use. These aspects, along with the considerable number of trials studying non-drug interventions, for which the definitions of phases do not apply, may contribute to the large proportion of trials lacking a phase classification. In addition, pediatric trial designs often rely on age-staggering and dose-finding approaches, based on pharmacokinetic and pharmacodynamic information, with ensuing within-trial adjustments. We found that earlier- and multi-phase trials, which likely incorporate some of these specific considerations of pediatric drug development, had higher rates of DMC adoption.

However, our results also highlighted the heterogeneity in the adoption (or reporting) of DMCs across pediatric trials. While trials on mostly novel advanced therapies, including genetic interventions, had the highest proportion of DMC use, few trials with devices reported DMCs, which highlights disparities across types of interventions, compounded by differences between clinical areas. Although the difference between NIH-funded and industry-funded trials is remarkable, it is not surprising considering DMCs have long been a standard component of trials sponsored by NIH institutes.^1^ Furthermore, it is noteworthy that most of the trials that were halted prematurely did not report to have established a DMC, in contrast with guidance on the relevance of DMCs for studies that plan for interim analysis for efficacy, futility, and/or harm. This is explained, to some extent, by the finding that most pediatric trials are terminated for reasons other than accumulated data from the trial, particularly low accrual rates, consistent with previous research.^11,16^

A previous proposal put forth a minimal set of reporting parameters on DMC implementation to support an update of the CONSORT statement for standards of reporting clinical trials.^6^ However, the latest version of the CONSORT statement only includes an item on interim analysis, overlooking details on DMCs or early termination of clinical trials.^17^ Similarly, we advocate that the data element on DMC adoption should be a mandatory field for completion of registration in ClinicalTrials.gov and that the availability of detailed information on DMC implementation should be encouraged for the sake of transparency, accountability, and quality of methodological features reported in trial registries. This would also facilitate and improve methodological research assessing the performance of DMCs and their impact on the validity of trial results. Although, the Data Monitoring Committees: Lessons, Ethics, Statistics (DAMOCLES) Study Group has established a widely used standard operating procedure to systematically describe the structure and organization of a DMC,^18^ the inclusion of a minimal set of descriptive parameters about DMC use in the clinical study protocol, trial reports publications, and trial registries, as previously advocated,^6^ is still lacking. The implementation of such proposals would enhance crucial methodological research on DMCs, which would ultimately provide insights into DMC performance and potential improvements to ensure more robust oversight in clinical trials.

### Limitations

A major limitation of our study relates to the results being based solely on the information provided in the protocol registration, which may not necessarily accurately reflect whether DMCs were actually implemented in practice. Despite the clarity of the item on registration, we cannot exclude misinterpretation of what a DMC is, or what the item refers to. The definitions of DMC are not always consistent, and the terminology that is used is highly heterogeneous, which may result in confusion with other committees with oversight or monitoring responsibilities such as Safety Monitoring Committees or Institutional Review Boards.^19^ In addition, around 11% of identified pediatric trial records did not answer on DMC adoption, as this is not a required field for completion of registration. We decided not to omit these from our analysis based on the judgment that omission would result in an overestimation of DMC adoption. The relatively low number of completion for this item limits the ability to generalize conclusions. Future research could focus on reviewing trial protocols and trial publications to evaluate the validity of these results. Conversely, we consider this to be a useful undesired result, as it draws attention to the need for improving the quality and completeness of registry data and reinforces recommendations on transparency of DMC operations.^20^ Evidence on the accuracy of reporting on trial registries is still limited and some data quality issues have been raised recently.^21–24^ Thus, the use of the DMC reporting item as a measurement of actual DMC implementation is not certain, even though legal requirements have been established for reporting of some trial methodological characteristics.^25^ Quality control review is provided, but it cannot ensure the veracity of the submitted information, nor determine perfect compliance with policies and legal requirements.^26^

An additional limitation relates to the comprehensiveness and generalizability of the data. Even though most of the included trials are completed, the vast majority did not report their results on ClinicalTrials.gov. Compliance with the FDAAA final rule has been shown to be poor.^27^ While ClinicalTrials.gov is the largest registry of clinical trials, some are solely registered in other registries such as the European registry or other national databases. Despite that recent trials are generally more likely to be required to report results, the observed trend is that older trials were more likely to have reported these data. For these reasons, we do not expect this study to be necessarily representative of the global scenario on DMC use in pediatric trials. Moreover, not all trials are registered,^28,29^ and trial registration has been previously associated with a lower risk of bias.^30^ Therefore, meta-epidemiological research based on registry records or results may be biased for higher-quality trials.

## Conclusion

Our analysis of registry records of pediatric RCTs revealed a higher frequency of DMC adoption than previously reported in reviews of published trial reports. However, the use of DMCs varied across key clinical and methodologic trial characteristics based on which their use is recommended. DMCs may still be underutilized in pediatric clinical trials. Moreover, evidence is still lacking on the quality of DMC performance and on the impact of DMC use, precluding the assessment of whether DMCs are being used optimally. Recommendations for reporting a minimal set of DMCs features and procedures in clinical trial registries should be implemented to promote transparency and accountability, and ultimately contribute to more robust oversight in clinical trials.

## Supplemental Material

sj-docx-1-ctj-10.1177_17407745231182417 – Supplemental material for Data monitoring committees in pediatric randomized controlled trials registered in ClinicalTrials.gov Click here for additional data file. Supplemental material, sj-docx-1-ctj-10.1177_17407745231182417 for Data monitoring committees in pediatric randomized controlled trials registered in ClinicalTrials.gov by Tiago Machado, Beatrice Mainoli, Daniel Caldeira, Joaquim J Ferreira and Ricardo M Fernandes in Clinical Trials

sj-xlsx-2-ctj-10.1177_17407745231182417 – Supplemental material for Data monitoring committees in pediatric randomized controlled trials registered in ClinicalTrials.gov Click here for additional data file. Supplemental material, sj-xlsx-2-ctj-10.1177_17407745231182417 for Data monitoring committees in pediatric randomized controlled trials registered in ClinicalTrials.gov by Tiago Machado, Beatrice Mainoli, Daniel Caldeira, Joaquim J Ferreira and Ricardo M Fernandes in Clinical Trials
