# Supplementary material for: Data monitoring committees in pediatric randomized controlled trials registered in ClinicalTrials.gov
Source: Clin Trials. 2023 Jun 27;20(6):624–31. doi: 10.1177/17407745231182417 (PMC10638853; doi:10.1177/17407745231182417)
Supplement: sj-docx-1-ctj-10.1177_17407745231182417 – Supplemental material for Data monitoring committees in pediatric randomized controlled trials registered in ClinicalTrials.gov [file sj-docx-1-ctj-10.1177_17407745231182417.docx]

| **Table S1 - Definitions and performed categorizations of ClinicalTrials.gov data elements** | | | |
| --- | --- | --- | --- |
| **ClinicalTrials.gov data element** | **Definition (as defined in ClinicalTrials.gov)** | **Categorization** | **Output data element** |
| Data Monitoring Committee | Whether a data monitoring committee has been appointed for this study. The data monitoring committee (board) is a group of independent scientists who are appointed to monitor the safety and scientific integrity of a human research intervention, and to make recommendations to the sponsor regarding the stopping of the trial for efficacy, for harms or for futility. The composition of the committee is dependent upon the scientific skills and knowledge required for monitoring the particular study. | No categorizations were performed. | Data Monitoring Committee |
| Primary Disease or Condition Being Studied in the Trial, or the Focus of the Study | The name(s) of the disease(s) or condition(s) studied in the clinical study, or the focus of the clinical study. | Each primary disease or condition were grouped according to second-level categories in the MeSH descriptor hierarchy. | Primary therapeutic area |
| Overall Recruitment Status | The recruitment status for the clinical study as a whole, based upon the status of the individual sites. If at least one facility in a multi-site clinical study has an Individual Site Status of "Recruiting," then the Overall Recruitment Status for the study must be "Recruiting."   - Not yet recruiting: Participants are not yet being recruited - Recruiting: Participants are currently being recruited, whether or not any participants have yet been enrolled - Enrolling by invitation: Participants are being (or will be) selected from a predetermined population - Active, not recruiting: Study is continuing, meaning participants are receiving an intervention or being examined, but new participants are not currently being recruited or enrolled - Completed: The study has concluded normally; participants are no longer receiving an intervention or being examined (that is, last participant’s last visit has occurred) - Suspended: Study halted prematurely but potentially will resume - Terminated: Study halted prematurely and will not resume; participants are no longer being examined or receiving intervention - Withdrawn: Study halted prematurely, prior to enrollment of first participant | ‘Ongoing’ status category was generated by combining ‘Active, not recruiting’, ‘Enrolling by invitation’, ‘Recruiting’ and ‘Not yet recruiting’ statuses. ‘Halted prematurely’ status category was generated by combining ‘Suspended’ and ‘Terminated’ status. ‘Withdrawn’, ‘Completed’ and ‘Unknown status’ categories were not transformed. | Completion status |
| Why Study Stopped | A brief explanation of the reason(s) why such clinical study was stopped (for a clinical study that is "Suspended," "Terminated," or "Withdrawn" prior to its planned completion as anticipated by the protocol). | Two authors independently categorized the information provided into one of the following categories:  1. Scientific data from the trial   - 1a. Unfavorable benefit-to-risk - 1b. Favorable benefit-to-risk - 1c. Other or unspecified benefit-to-risk   2. Other than scientific data from the trial   - 2a. Insufficient accrual rate - 2b. Unspecified business decision/strategic reason - 2c. Trial administration or conduct (issues with protocol, site, etc.) - 2d. External information (results from other trials, competing trials, or changes in standard of care) - 2e. Funding - 2f. Product withdrawal - 2g. Lack of drug supply (other than drug withdrawal) - 2h. Trial personnel issues (investigators, staff, etc.) - 2i. Issues related to the coronavirus pandemic - 2j. Other (e.g., uninformative or non-specific text)   3. Termination Reason Not Provided | Reason for halting prematurely |
| Facility Information | For each participating facility in a clinical study, the following information is available: Facility Name; City; State/Province; ZIP/Postal Code; Country | The number of distinct countries of study sites per trial was assessed as a dichotomous variable: single-country or multinational trials. | Study sites (number of countries) |
|  |  | The number of studies sites per trial was assessed as a dichotomous variable: single-center or multicenter trials. | Study sites (number of sites) |
| Age limits | The minimum and maximum age of potential participants eligible for the clinical study, provided in relevant units of time. | Age limits were categorized into six age groups: newborns (0-27 days); infants (28 days – 12 months); toddlers (12 months – 2 years); 2- to 5-years-old children; 6- to 11-years-old children; 12- to 18-years-old. | Age groups |
| Funder type (sponsor, collaborators) | Describes the organization that provides funding or support for a clinical study. This support may include activities related to funding, design, implementation, data analysis, or reporting. Organizations listed as sponsors and collaborators for a study are considered the funders of the study. ClinicalTrials.gov refers to four types of funders:   - U.S. National Institutes of Health - Other U.S. Federal agencies (for example, Food and Drug Administration, Centers for Disease Control and Prevention, or U.S. Department of Veterans Affairs) - Industry (for example: pharmaceutical and device companies) - All others (including individuals, universities, and community-based organizations) | We classified as an industry-funded trial if ‘Industry’ was listed as the funder type of the lead sponsor or a collaborator without U.S. National Institutes of Health (NIH) as the lead sponsor or collaborator. We classified as NIH-funded for those for which NIH was the lead sponsor or a collaborator with a non-industry lead sponsor. All other trials were considered as ‘Other’ regarding the source of funding. | Funding |
| Study Phase | The stage of a clinical trial studying a drug or biological product, based on definitions developed by the U.S. Food and Drug Administration (FDA). The phase is based on the study's objective, the number of participants, and other characteristics. There are five phases: Early Phase 1 (formerly listed as Phase 0), Phase 1, Phase 2, Phase 3, and Phase 4. Not Applicable is used to describe trials without FDA-defined phases, including trials of devices or behavioral interventions.   - Early Phase 1 (Formerly listed as "Phase 0"): Exploratory trials, involving very limited human exposure, with no therapeutic or diagnostic intent (e.g., screening studies, microdose studies). - Phase 1: Includes initial studies to determine the metabolism and pharmacologic actions of drugs in humans, the side effects associated with increasing doses, and to gain early evidence of effectiveness; may include healthy participants and/or patients. - Phase 1/Phase 2: Trials that are a combination of phases 1 and 2. - Phase 2: Includes controlled clinical studies conducted to evaluate the effectiveness of the drug for a particular indication or indications in participants with the disease or condition under study and to determine the common short-term side effects and risks. - Phase 2/Phase 3: Trials that are a combination of phases 2 and 3. - Phase 3: Includes trials conducted after preliminary evidence suggesting effectiveness of the drug has been obtained, and are intended to gather additional information to evaluate the overall benefit-risk relationship of the drug. - Phase 4: Studies of FDA-approved drugs to delineate additional information including the drug's risks, benefits, and optimal use. | We grouped ‘Early Phase 1’ and ‘Phase 1’ into ‘Phase 1’ and ‘Phase 1/Phase 2’ and ‘Phase 2/Phase 3’ into ‘Multiphase’. Other categories remained as defined in ClinicalTrials.gov. | Clinical research phase |
| Intervention Type | A process or action that is the focus of a clinical study. Interventions include:   - Drug: Including placebo - Device: Including sham - Biological/Vaccine - Procedure/Surgery - Radiation - Behavioral: For example, psychotherapy, lifestyle counseling - Genetic: Including gene transfer, stem cell and recombinant DNA - Dietary Supplement: For example, vitamins, minerals - Combination Product: Combining a drug and device, a biological product and device; a drug and biological product; or a drug, biological product, and device - Diagnostic Test: For example, imaging, in-vitro - Other | No categorizations were performed. | Type of intervention |
| Arm Type | The role of each arm in the clinical trial:   - Experimental - Active Comparator - Placebo Comparator - Sham Comparator - No Intervention - Other | Trials reporting at least one arm classified as ‘Placebo Comparator’ or ‘Sham Comparator’ were grouped as ‘Placebo-controlled trials’. | Placebo control |
| Primary purpose | The main objective of the intervention(s) being evaluated by the clinical trial.   - Treatment: One or more interventions are being evaluated for treating a disease, syndrome, or condition. - Prevention: One or more interventions are being assessed for preventing the development of a specific disease or health condition. - Diagnostic: One or more interventions are being evaluated for identifying a disease or health condition. - Supportive Care: One or more interventions are evaluated for maximizing comfort, minimizing side effects, or mitigating against a decline in the participant's health or function. - Screening: One or more interventions are assessed or examined for identifying a condition, or risk factors for a condition, in people who are not yet known to have the condition or risk factor. - Health Services Research: One or more interventions for evaluating the delivery, processes, management, organization, or financing of healthcare. - Basic Science: One or more interventions for examining the basic mechanism of action (for example, physiology or biomechanics of an intervention). - Device Feasibility: An intervention of a device product is being evaluated in a small clinical trial (generally fewer than 10 participants) to determine the feasibility of the product; or a clinical trial to test a prototype device for feasibility and not health outcomes. Such studies are conducted to confirm the design and operating specifications of a device before beginning a full clinical trial. - Other: None of the other options applies. | No categorizations were performed. | Primary purpose |
| Masking | The party or parties involved in the clinical trial who are prevented from having knowledge of the interventions assigned to individual participants.   - Roles, if Masking:   - Participant   - Care Provider   - Investigator   - Outcomes Assessor: The individual who evaluates the outcome(s) of interest - No Masking | No categorizations were performed. | Blinding |
| Total Number Affected by Any Serious Adverse Event | Overall number of participants affected by one or more Serious Adverse Events, for each arm/group. | For trials reporting safety results, this data element was dichotomized into trials reporting at least one serious adverse event, and trials reporting no serious adverse events. | Serious adverse events |
| Total Number Affected by All-Cause Mortality | Overall number of participants, in each arm/group, who died due to any cause. | For trials reporting safety results, this data element was dichotomized into trials reporting at least one death, and trials reporting no deaths. | All-cause mortality |
| Enrollment | The estimated total number of participants to be enrolled (target number) or the actual total number of participants that are enrolled in the clinical study. | This data element was dichotomized into trials with up to 100 participants and trials with 100 or more participants. For median sample size calculations, no categorizations were performed. | Sample size |

**Table S2 - Reported adoption of a DMC stratified by characteristics of the intervention**

|  |  | **Data monitoring committee – n (%)** | | |
| --- | --- | --- | --- | --- |
| **Intervention characteristics** | **Total - n (%)** | **DMC adoption** | **No DMC adoption** | **No answer regarding DMC** |
| **Type of intervention*** | | | | |
| Drug | 4,299 (30.9%) | 2,159 (50.2%) | 1,668 (38.8%) | 472 (11.0%) |
| Behavioral | 3,193 (22.9%) | 1,045 (32.7%) | 1,894 (59.3%) | 254 (8.0%) |
| Device | 1,573 (11.3%) | 575 (36.6%) | 838 (53.3%) | 160 (10.2%) |
| Dietary Supplement | 1,071 (7.7%) | 426 (39.8%) | 554 (51.7%) | 91 (8.5%) |
| Biological/Vaccine | 994 (7.1%) | 463 (46.6%) | 340 (34.2%) | 191 (19.2%) |
| Procedure/Surgery | 991 (7.1%) | 409 (41.3%) | 434 (43.8%) | 148 (14.9%) |
| Combination Product | 65 (0.5%) | 34 (52.3%) | 23 (35.4%) | 8 (12.3%) |
| Diagnostic Test | 68 (0.5%) | 18 (26.5%) | 39 (57.4%) | 11 (16.2%) |
| Radiation | 26 (0.2%) | 13 (50.0%) | 6 (23.1%) | 7 (26.9%) |
| Genetic | 16 (0.1%) | 12 (75.0%) | 3 (18.8%) | 1 (6.3%) |
| **Primary purpose** | | | | |
| Treatment | 7,714 (55.4%) | 3,325 (43.1%) | 3,540 (45.9%) | 849 (11%) |
| Prevention | 3,323 (23.9%) | 1,320 (39.7%) | 1,574 (47.4%) | 429 (12.9%) |
| Supportive Care | 965 (6.9%) | 315 (32.6%) | 566 (58.7%) | 84 (8.7%) |
| Health Services Research | 397 (2.9%) | 124 (31.2%) | 237 (59.7%) | 36 (9.1%) |
| Basic Science | 327 (2.3%) | 96 (29.4%) | 200 (61.2%) | 31 (9.5%) |
| Diagnostic | 203 (1.5%) | 62 (30.5%) | 118 (58.1%) | 23 (11.3%) |
| Screening | 70 (0.5%) | 17 (24.3%) | 45 (64.3%) | 8 (11.4%) |
| Device Feasibility | 13 (0.1%) | 4 (30.8%) | 8 (61.5%) | 1 (7.7%) |
| Other | 671 (4.8%) | 200 (29.8%) | 381 (56.8%) | 90 (13.4%) |
| No data regarding primary purpose | 245 (1.8%) | 66 (26.9%) | 158 (64.5%) | 21 (8.6%) |

* Non-mutually exclusive groups

**Table S3 - Reported adoption of a DMC stratified by year of registration**

|  |  | **Data monitoring committee – n (%)** | | |
| --- | --- | --- | --- | --- |
| **Year of registration** | **Total - n (%)** | **DMC adoption** | **No DMC adoption** | **No answer** |
| 2008 | 655 (4.7%) | 269 (41.1%) | 316 (48.2%) | 70 (10.7%) |
| 2009 | 659 (4.7%) | 272 (41.3%) | 320 (48.6%) | 67 (10.2%) |
| 2010 | 693 (5.0%) | 322 (46.5%) | 303 (43.7%) | 68 (9.8%) |
| 2011 | 701 (5.0%) | 318 (45.4%) | 328 (46.8%) | 55 (7.8%) |
| 2012 | 838 (6.0%) | 383 (45.7%) | 415 (49.5%) | 40 (4.8%) |
| 2013 | 811 (5.8%) | 358 (44.1%) | 418 (51.5%) | 35 (4.3%) |
| 2014 | 950 (6.8%) | 405 (42.6%) | 481 (50.6%) | 64 (6.7%) |
| 2015 | 1,047 (7.5%) | 427 (40.8%) | 583 (55.7%) | 37 (3.5%) |
| 2016 | 1,124 (8.1%) | 469 (41.7%) | 572 (50.9%) | 83 (7.4%) |
| 2017 | 1,184 (8.5%) | 506 (42.7%) | 554 (46.8%) | 124 (10.5%) |
| 2018 | 1,304 (9.4%) | 471 (36.1%) | 638 (48.9%) | 195 (15.0%) |
| 2019 | 1,247 (9.0%) | 420 (33.7%) | 608 (48.8%) | 219 (17.6%) |
| 2020 | 1,307 (9.4%) | 455 (34.8%) | 603 (46.1%) | 249 (19.1%) |
| 2021 | 1,408 (10.1%) | 454 (32.2%) | 688 (48.9%) | 266 (18.9%) |

**Table S4 - Reported adoption of a DMC stratified by primary therapeutic area being studied**

|  |  | **Data monitoring committee – n (%)** | | |
| --- | --- | --- | --- | --- |
| **Primary therapeutic areas*** | **Total - n (%)** | **DMC adoption** | **No DMC adoption** | **No answer** |
| Infections | 1,901 (13.6%) | 852 (44.8%) | 777 (40.9%) | 272 (14.3%) |
| Neoplasms | 234 (1.7%) | 134 (57.3%) | 79 (33.8%) | 21 (9.0%) |
| Musculoskeletal Diseases | 560 (4.0%) | 232 (41.4%) | 266 (47.5%) | 62 (11.1%) |
| Digestive System Diseases | 715 (5.1%) | 335 (46.9%) | 293 (41.0%) | 87 (12.2%) |
| Stomatognathic Diseases | 626 (4.5%) | 228 (36.4%) | 287 (45.8%) | 111 (17.7%) |
| Respiratory Tract Diseases | 1,653 (11.9%) | 719 (43.5%) | 726 (43.9%) | 208 (12.6%) |
| Otorhinolaryngologic Diseases | 283 (2.0%) | 85 (30.0%) | 159 (56.2%) | 39 (13.8%) |
| Nervous System Diseases | 2,315 (16.6%) | 980 (42.3%) | 1,069 (46.2%) | 266 (11.5%) |
| Eye Diseases | 323 (2.3%) | 158 (48.9%) | 120 (37.2%) | 45 (13.9%) |
| Urogenital Diseases | 761 (5.5%) | 366 (48.1%) | 320 (42.0%) | 75 (9.9%) |
| Cardiovascular Diseases | 509 (3.7%) | 292 (57.4%) | 174 (34.2%) | 43 (8.4%) |
| Hemic and Lymphatic Diseases | 359 (2.6%) | 190 (52.9%) | 130 (36.2%) | 39 (10.9%) |
| Congenital, Hereditary, and Neonatal Diseases and Abnormalities | 1,674 (12.0%) | 808 (48.3%) | 686 (41.0%) | 180 (10.8%) |
| Skin and Connective Tissue Diseases | 343 (2.5%) | 122 (35.6%) | 192 (56.0%) | 29 (8.5%) |
| Nutritional and Metabolic Diseases | 1,515 (10.9%) | 607 (40.1%) | 786 (51.9%) | 122 (8.1%) |
| Endocrine System Diseases | 414 (3.0%) | 175 (42.3%) | 214 (51.7%) | 25 (6.0%) |
| Immune System Diseases | 1,151 (8.3%) | 459 (39.9%) | 576 (50.0%) | 116 (10.1%) |
| Pathological Conditions, Signs and Symptoms | 3,539 (25.4%) | 1,547 (43.7%) | 1,676 (47.4%) | 316 (8.9%) |
| Chemically-Induced Disorders | 194 (1.4%) | 88 (45.4%) | 100 (51.5%) | 6 (3.1%) |
| Wounds and Injuries | 454 (3.3%) | 165 (36.3%) | 253 (55.7%) | 36 (7.9%) |
| Behavior and Behavior Mechanisms | 460 (3.3%) | 205 (44.6%) | 216 (47.0%) | 39 (8.5%) |
| Mental Disorders | 1,834 (13.2%) | 714 (38.9%) | 958 (52.2%) | 162 (8.8%) |
| No data regarding condition | 2,721 (19.5%) | 1,601 (58.8%) | 347 (12.8%) | 773 (28.4%) |

* Non-mutually exclusive groups

**Table S5 - Reported adoption of a DMC stratified by blinding of involved parties**

|  |  | **Data monitoring committee – n (%)** | | |
| --- | --- | --- | --- | --- |
| **Trial characteristics** | **Total - n (%)** | **DMC adoption** | **No DMC adoption** | **No answer** |
| **Blinding of participants** | | | | |
| Blinded | 6,320 (45.4%) | 2,802 (44.3%) | 2,731 (43.2%) | 787 (12.5%) |
| Non-blinded | 7,608 (54.6%) | 2,727 (35.8%) | 4,096 (53.8%) | 785 (10.3%) |
| **Blinding of caregivers** | | | | |
| Blinded | 4,084 (29.3%) | 2,023 (49.5%) | 1,664 (40.7%) | 397 (9.7%) |
| Non-blinded | 9,844 (70.7%) | 3,506 (35.6%) | 5,163 (52.4%) | 1,175 (11.9%) |
| **Blinding of investigators** | | | | |
| Blinded | 5,134 (36.9%) | 2,433 (47.4%) | 2,194 (42.7%) | 507 (9.9%) |
| Non-blinded | 8,794 (63.1%) | 3,096 (35.2%) | 4,633 (52.7%) | 1,065 (12.1%) |
| **Blinding of outcomes assessors** | | | | |
| Blinded | 5,964 (42.8%) | 2,706 (45.4%) | 2,625 (44.0%) | 633 (10.6%) |
| Non-blinded | 7,964 (57.2%) | 2,823 (35.4%) | 4,202 (52.8%) | 939 (11.8%) |

**Table S6 – Reasons for prematurely halting of trials**

|  |  | **Data monitoring committee – n (%)** | | |
| --- | --- | --- | --- | --- |
| **Trial characteristics** | **Total - n (%)** | **DMC adoption** | **No DMC adoption** | **No answer** |
| **1. Scientific data from the trial** | **78 (11.4%)** | **49 (15.7%)** | **24 (7.3%)** | **5 (11.9%)** |
| 1a. Unfavorable benefit-to-risk | 55 (8.1%) | 34 (10.9%) | 17 (5.2%) | 4 (9.5%) |
| 1b. Favorable benefit-to-risk | 6 (0.9%) | 4 (1.3%) | 2 (0.6%) | 0 (0%) |
| 1c. Other or unspecified benefit-to-risk | 17 (2.5%) | 11 (3.5%) | 5 (1.5%) | 1 (2.4%) |
| **2. Other than scientific data from the trial** | **555 (81.4%)** | **244 (78%)** | **279 (85.3%)** | **32 (76.2%)** |
| 2a. Insufficient accrual rate | 277 (40.6%) | 119 (38%) | 152 (46.5%) | 6 (14.3%) |
| 2b. Unspecified business decision/strategic reason | 35 (5.1%) | 16 (5.1%) | 16 (4.9%) | 3 (7.1%) |
| 2c. Trial administration or conduct (issues with protocol, site, etc.) | 68 (10%) | 30 (9.6%) | 29 (8.9%) | 9 (21.4%) |
| 2d. External information (results from other trials, competing trials, or changes in standard of care) | 28 (4.1%) | 17 (5.4%) | 9 (2.8%) | 2 (4.8%) |
| 2e. Funding | 31 (4.5%) | 15 (4.8%) | 15 (4.6%) | 1 (2.4%) |
| 2f. Product withdrawal | 14 (2.1%) | 8 (2.6%) | 5 (1.5%) | 1 (2.4%) |
| 2g. Lack of drug supply (other than drug withdrawal) | 8 (1.2%) | 3 (1%) | 4 (1.2%) | 1 (2.4%) |
| 2h. Trial personnel issues (investigators, staff, etc.) | 32 (4.7%) | 13 (4.2%) | 17 (5.2%) | 2 (4.8%) |
| 2i. Coronavirus pandemic related issues | 49 (7.2%) | 17 (5.4%) | 26 (8%) | 6 (14.3%) |
| 2j. Other (e.g., uninformative or non-specific text) | 13 (1.9%) | 6 (1.9%) | 6 (1.8%) | 1 (2.4%) |
| **3. Stopping reason not provided** | **49 (7.2%)** | **20 (6.4%)** | **24 (7.3%)** | **5 (11.9%)** |
| **Total trials halted prematurely** | **682 (100%)** | **313 (100%)** | **327 (100%)** | **42 (100%)** |

Percentages represent proportions for each column total.

**Figure S1. Registered trials per year**

**
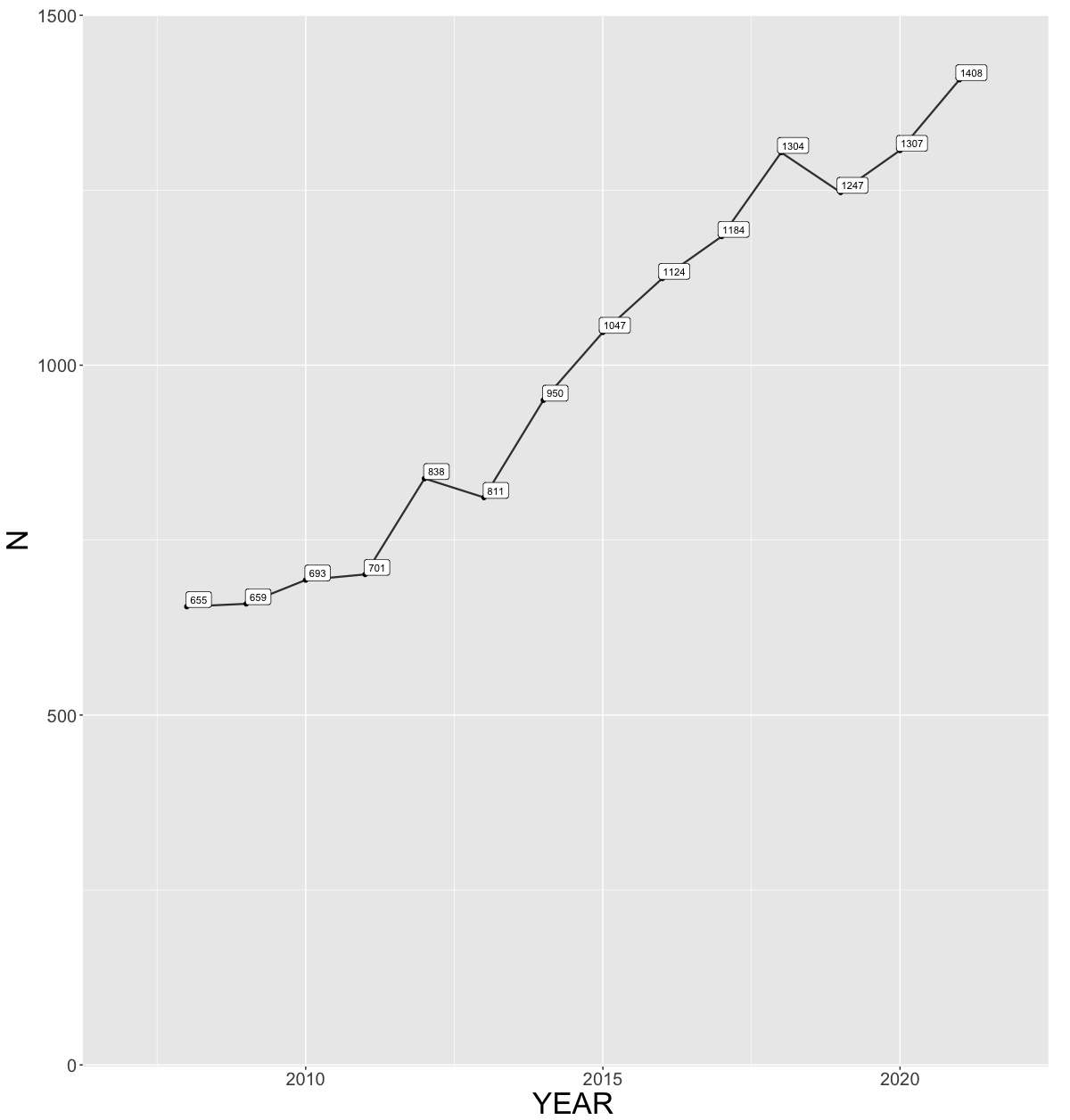
**

**Figure S2. Violin plot comparing sample size of trials with a DMC (green) and trials without a DMC (red).**


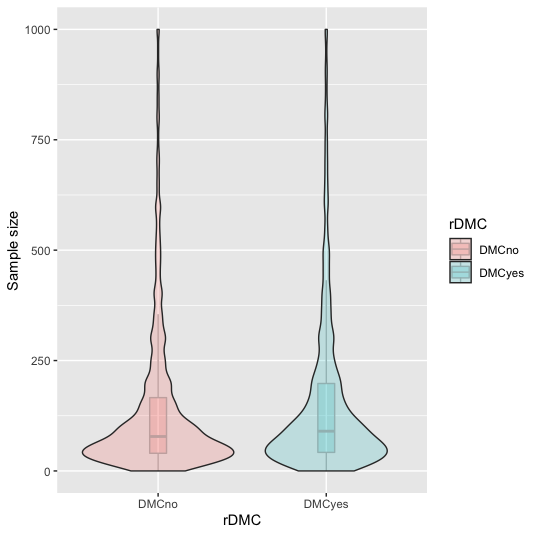


Graph truncated for clarity.
